# Artificial Intelligence in Renal Transplantation Over the Past Decade: A Narrative Review of Clinical Applications, Current Limitations, and Future Directions

**DOI:** 10.7759/cureus.100134

**Published:** 2025-12-26

**Authors:** Ahmed Anber, Youssef Mohamed, Aryan Maleki, Sami Atiq, Larisa Radu, Ibrahim Omar, Abdelrahman Sayed

**Affiliations:** 1 Urology, Barking Havering and Redbridge NHS Trust, London, GBR; 2 Urology, Cambridge University Hospitals NHS Trust, Cambridge, GBR; 3 Urology, Addenbrooke’s Hospital, Cambridge University Hospitals NHS Trust, Cambridge, GBR; 4 Urology, Hereford County Hospital, Wye Valley NHS Trust, Hereford, GBR; 5 Trauma and Orthopedics, Princess Royal University Hospital, King's College NHS Foundation Trust, London, GBR; 6 Orthopedics and Trauma, King’s College University Hospital, London, GBR; 7 Urology, Great Western Hospital, Swindon, GBR; 8 Trauma and Orthopedics, Cardiff University Hospitals, Cardiff, GBR

**Keywords:** ai, artificial intelligence(ai), ml, renal transplantation, urology

## Abstract

This narrative review examines the use of artificial intelligence (AI) and machine learning (ML) in kidney transplantation (KT) during the past 10 years, highlighting advancements in clinical applications and future potential. In pretransplant settings, AI algorithms assist in matching donors with recipients and predicting survival outcomes, aiming to reduce organ discard rates and improve allocation efficiency beyond traditional scoring systems like the Kidney Donor Profile Index. Surgical data science utilizes AI to enhance robotic surgery through augmented reality for real-time anatomical visualization and 3D printed models for preoperative planning. Furthermore, ML is applied to assess organ quality during normothermic machine perfusion. Regarding post-transplant outcomes, artificial neural networks have demonstrated superior accuracy in predicting graft survival and rejection compared to conventional statistical methods. Despite these advancements, clinical application is hindered by limitations such as overfitting, selection bias from single-center data, and a lack of external validation.

## Introduction and background

Kidney transplantation (KT) stands as the optimal therapeutic strategy for individuals afflicted with end-stage renal disease. This procedure is instrumental in not only extending survival but also in mitigating cardiovascular complications and significantly enhancing the overall quality of life for these patients [1,2]. Delayed-graft function (DGF) is defined as acute kidney injury (AKI) following a kidney transplant (KTx) that necessitates at least one dialysis treatment within the initial week post-operation. This condition leads to extended hospital stays, an increased incidence of acute rejection, and consequently, a reduction in the long-term survival of the transplanted organ [3-5].

Artificial intelligence (AI) has transcended its general utility to become a cornerstone of modern medicine, particularly within the field of transplantation. By enabling systems to simulate intelligent and cognitive functions, AI encompasses a diverse array of applications. Within this framework, machine learning (ML) has emerged as a critical subset, gaining substantial traction in healthcare due to the synergistic advancements in big data analytics and computational power [6]. ML functions by systematically analyzing datasets to discern patterns and predict outcomes based on historical precedents. Through this process, the algorithm iteratively refines its internal structure and governing rules to optimize performance [7-9]. The increased availability of numerical data and the capacity of AI algorithms to process and learn from extensive datasets have led to the widespread application of AI in various medical fields, including clinical decision-making, biomedical research, and medical education [10].

AI is employed across a wide spectrum of studies within KT, extending from detailed pathological evaluations to comprehensive predictions of transplant outcomes [11-16]. In this narrative review article, we aim to define the key applications of AI in KT over the last decade, summarize the current limitations associated with the integration of these technologies, and outline prospective future directions in the field.

## Review

Pretransplant matching

Candidates waiting for a deceased-donor kidney transplant (DDKT) face a significant delay, with a median wait time of 7.6 years, due to the critical scarcity of available organs [17]. Nevertheless, 50% of the kidneys recovered from marginal donors, those who are older or have underlying health issues, are ultimately discarded instead of being used for transplantation [18]. Receiving a KTx using a marginal kidney has been shown in earlier research to significantly improve patient survival compared to staying on the waiting list [19,20].

The KTx procedure starts with the offering of an organ, and the choice to either accept or reject this offer is critical. Not every patient awaiting a KTx will benefit adequately from every available donated kidney, particularly those from an expanded criteria donor (ECD) [21]. To enhance the process of organ distribution and reduce the number of discarded organs, several decision-support tools have been developed. A notable example is the Kidney Donor Risk Index (KDRI), established in 2009. This index combines 14 different factors about the donor and the transplantation procedure into a single score, which is used to predict the risk of the transplanted kidney failing after a KTx [22]. 

The KDRI suggests that KTxs with a high index (above 1.45) have worse five-year graft survival. Building on this, the Kidney Donor Profile Index (KDPI) was created, which focuses only on donor characteristics and is reported as a percentile. Kidneys classified as "high KDPI" (85% or greater) are associated with lower five-year survival and a higher chance of graft failure compared to those with a KDPI under 85% [23]. 

The Estimated Post-Transplant Survival Score (EPTS) was developed to optimize donor-recipient matching. This numerical score uses four recipient-specific factors (age, duration of dialysis, pre-existing diabetes, and prior solid-organ transplantation) to predict survival following transplantation and guide kidney distribution. Both the KDPI and the EPTS were integrated into the kidney allocation system (KAS) in 2014 by the United Network for Organ Sharing (UNOS). Currently, the system prioritizes allocating the highest quality kidneys (those with a KDPI below 20) to recipients with the lowest expected post-transplant survival risk (an EPTS of 20 or less) [24,25].

While scoring systems like the KDPI aim to objectively identify high-risk but still usable kidneys, there was concern that their introduction might increase organ discards. However, a study by Bae et al. found that the overall discard rate remained statistically similar before (18.1%) and after (18.3%) KDPI implementation. Interestingly, the study did reveal an increased discard likelihood for one specific subset: standard criteria donor (SCD) kidneys that had a high KDPI score (greater than 85), suggesting an increased possibility of discard when the older ECD classification and the newer KDPI score conflicted [18].

AI algorithms can also help streamline organ allocation by examining past data and current information. This allows transplant centers to make more informed choices about organ distribution, considering variables like location, transport challenges, and patient characteristics [24].

Bae et al. developed a predictive tool to estimate survival outcomes following DDKT by analyzing the interaction between donor quality (KDPI) and candidate condition (EPTS). Studying over 120000 recipients and 376000 waitlisted candidates, the researchers found that while high-risk candidates (EPTS >40) derived substantial survival benefit even from marginal kidneys (KDPI 100), low-risk candidates experienced limited benefit. This ML-based tool aids individualized clinical decision-making regarding the acceptance of marginal kidney offers [26]. 

Brown et al. utilized Bayesian belief network (BBN) modeling to predict kidney graft survival based on pretransplant variables. Retrospectively analyzing 5144 deceased donor kidney recipients from the US Renal Data System (2000-2001), the researchers constructed a network of 48 clinical variables. The model, validated on an external cohort of 2204 patients, predicted graft failure within one or three years with an area under the curve (AUC) of 0.63 and 80% specificity. Key predictors included recipient BMI, gender, race, and donor age, while human leukocyte antigen (HLA) matching showed a weaker association [27].

Kilambi et al. introduced a decision-tree methodology to aid individual-level decision-making in KT. Addressing the dilemma of accepting a current marginal kidney offer versus waiting for a potentially better one, the authors developed a tool that calculates the survival benefit of each choice. The model incorporates patient and donor characteristics, transplant center performance, and individual utility preferences. Tested on a dataset of 1,000 deceased-donor kidney offers from 2016, the tool demonstrated 61% accuracy in predicting optimal acceptance decisions when evaluating up to one year of future offers. The study highlights that personalized, quantitative assessments can mitigate risk aversion in accepting marginal kidneys, potentially reducing discard rates and improving patient survival [28].

AI-assisted surgery

Surgical data science (SDS) is an area that uses AI and ML to improve surgical procedures, education, and patient outcomes. Its goal is to employ sophisticated computational methods to better various facets of surgery, including planning before an operation, making choices, and providing assistance during the surgery [29].

AI improves robotic KT surgery by offering better control over the surgical tools, enhancing accuracy and stability, and providing the surgeon with immediate operational data. Furthermore, AI-powered imaging, such as augmented reality (AR), assists in visualizing the operative area optimally by layering digital information onto the real-time surgical view. Specifically, 3D AR guidance can direct the surgeon by processing images to highlight crucial anatomical features, recommend the best places for incisions, and anticipate possible issues [30,31].

AI technologies provide support for decisions in the operating room; AI systems constantly monitor procedures and a patient's vital signs. Real-time analysis of this data assists in forecasting the procedure's duration to improve scheduling and resource allocation, as well as detecting irregularities and offering immediate support to the surgical team [29].

Smart Digital Surgery (SDS) has the potential to transform surgical training, especially for KTxs, by using AI-driven simulation systems to offer highly realistic and engaging practice. A specific SDS approach, Objective Computer-Aided Skill Evaluation of Surgical Technical Skill (OCASE-T), employs computational analysis of data gathered from motion tracking, video footage, and instrument sensors to provide a systematic, objective, impartial, and economical assessment of a trainee's surgical skills [32].

AI uses imaging data to create detailed 3D anatomical models of the patient. These models can be 3D printed for use in pre-operative planning and surgical simulation. Practicing the surgery on these models allows surgeons to anticipate challenges and refine their techniques before the actual operation [33].

Sommer et al. developed a functional classification system for ex vivo kidneys during normothermic machine perfusion (NMP) using hyperspectral imaging (HSI) and convolutional neural networks (CNN). Analyzing 26 kidneys, the researchers utilized HSI in the 550-995 nm range to extract spectral data, which was then processed by a ResNet-18-based architecture (KidneyResNet) to predict inulin clearance, a marker of glomerular filtration rate. The model achieved 84% accuracy on individual regions of interest (ROIs) and improved to 96% accuracy when using a majority decision across all ROIs per kidney in the validation set. This approach demonstrates the potential of non-invasive, objective optical imaging to assess organ quality prior to transplantation [34].

**Figure 1:**
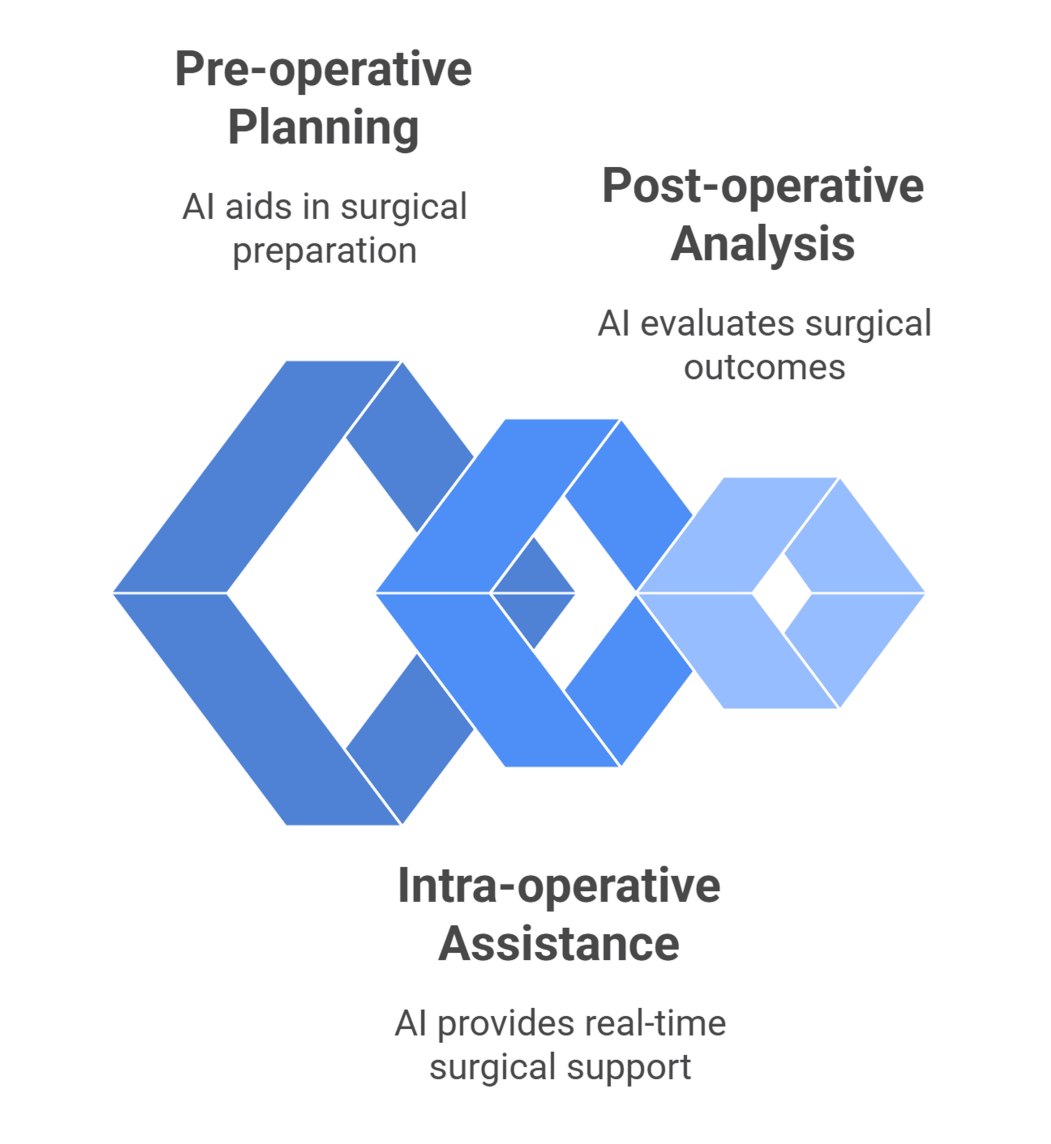
AI-assisted surgery in renal transplantation Figure created by the authors based on sources [29-31].

Zaza et al. employed comparative proteomics and ML to evaluate the biological effects of NMP on marginal donor kidneys. Analyzing tissue and urine samples from eight discarded kidneys subjected to 120 minutes of NMP, the study identified distinctive protein signatures, notably the upregulation of Latexin and mitochondrial proteins, alongside the downregulation of complement and coagulation factors. These findings indicate that NMP actively reconditions organs by enhancing metabolic activity and reducing inflammatory markers, thereby supporting its potential to rehabilitate extended criteria grafts for transplantation (Figure 1) [35].

Prediction of rejection and graft survival

An artificial neural network (ANN), trained on data from 27 patients and 33 variables, was employed by Simic-Ogrizovic et al. to forecast the advancement of chronic rejection. Their findings indicated that the ANN was a more dependable predictor of the chronic rejection's trajectory compared to standard statistical approaches [36].

Lin et al. compared single-time-point prediction models (logistic regression (LR) and single-output ANNs) with multiple time-point models (Cox models and multiple-output ANNs) for predicting KTx outcomes. The study found that both single and multiple time-point models offered similar AUC performance, although multiple-output ANNs performed worse when many observations were censored. Furthermore, the researchers noted that LR could perform as well as ANNs unless the relationship between predictors and outcomes involved strong interactions or nonlinear patterns [37]. In a separate investigation, a study developed an ANN to predict five-year graft survival rates specifically for living-donor kidney transplant recipients. The ANN model's performance was compared to predictions from nomograms based on Cox regression. The model was trained using data from 1581 patients and validated with 319 patients. The ANNs demonstrated a significantly higher positive predictive value for graft survival, reaching 82.1% compared to only 43.5% for the Cox regression-based nomogram. Therefore, the researchers concluded that ANNs provide superior accuracy and sensitivity in forecasting five-year graft survival when benchmarked against the conventional Cox regression-based nomogram [38].

Using United States Renal Data System information, Tang et al. evaluated the three-year graft survival rate for KTx patients with systemic lupus erythematosus (SLE). They employed classification trees, LR, and ANNs for their predictive modeling. The discriminative power of these models was quantified by the 95% confidence interval of the area under the receiver-operator characteristic curve (AUROC). The study concluded that the simpler models, LR and classification trees, achieved similar performance to the more complex ANNs [39].

Zhou et al. utilized a least absolute shrinkage and selection operator (LASSO) machine-learning algorithm within a Cox proportional hazards model to identify a protein signature predicting post-transplant renal graft survival. Motivated by the limitations of serum creatinine and data from a pilot study of 47 renal transplant recipients, the researchers analyzed 17 proteins previously associated with rejection. The variable selection process successfully identified kidney injury molecule-1 (KIM-1) and vascular endothelial growth factor receptor 2 (VEGF-R2) as critical biomarkers significantly associated with the hazard of allograft loss. This study demonstrates the efficacy of regularized regression methods in selecting sparse, predictive signals from high-dimensional proteomic data [40].

Mark et al. developed an ensemble ML model to predict KTx survival, aiming to surpass the accuracy of the Estimated Post Transplant Survival (EPTS) score currently used in US allocation. By analyzing UNOS data from 2002 to 2011, the researchers combined random survival forests with Cox proportional hazards models, stratifying recipients by age (≤50 and >50 years) to tailor variable selection. This ensemble approach achieved a five-year concordance index (C-index) of 0.724, significantly outperforming the EPTS model's 0.697. The study highlights how integrating diverse statistical methods can better capture complex interactions, potentially improving allocation efficiency and patient counseling [41].

Quinino et al. developed an ML model to predict immediate graft function (IGF) in DDKT recipients. Analyzing data from 859 unsensitized patients, the researchers compared seven algorithms, including XGBoost, Logistic Regression, and Random Forest. The eXtreme Gradient Boosting (XGBoost) model demonstrated superior performance, achieving an AUC of 0.78, with a specificity of 0.78 and sensitivity of 0.64. This predictive tool could optimize resource allocation by identifying patients most likely to benefit from interventions such as machine perfusion [42].

Limitations and future directions

AI in nephrology and renal transplantation faces substantial limitations that currently preclude fully autonomous clinical use, despite promising early results. These constraints span data quality, model performance, interpretability, ethical-legal issues, and practical integration into transplant workflows [43-45].

Most AI models in KT are trained on retrospective, single-center, or few-center datasets that are heterogeneous, incomplete, and variably coded, which undermines robustness and external validity. Many studies lack rigorous external validation or are tested only in simulation, so performance may drop sharply when deployed across different populations, donor types, and healthcare systems. Limited availability of high-quality, longitudinal transplant registries and systematic biopsy, imaging, and omics data further constrains the development of generalizable models for rejection, graft survival, and immunosuppression tailoring [45,46].

AI has been proven effective and is now a standard tool for various tasks across many areas of medicine [10,47]. Nephrology is well-suited to benefit from advancements in AI because patients require monitoring over many decades. Standardized practices, established through widely accepted recommendations and consensus, already exist across all areas of nephrology, including dialysis, KT, and general clinical practice. Furthermore, in many nations, the field of nephrology has been utilizing digital systems for over two decades [48].

ML models show promise in transplantology but face critical limitations in clinical application. Overfitting is a primary concern, where complex models memorize noise in the training data, leading to excellent performance on training sets but poor reliability when predicting outcomes for new, unseen patients. This issue is amplified by reliance on retrospective data often gathered from a single institution or a limited patient group, introducing selection bias and hindering the model's ability to generalize across the diverse patient populations and clinical settings encountered in the real world. A lack of robust external validation across different environments further casts doubt on the practical clinical utility of these models [43].

AI's future role in KT is exceptionally promising and could revolutionize the entire process. Integrating AI into patient registration systems will simplify information management, enabling real-time data tracking and analysis. Advanced AI surveillance, building upon current databases, can monitor patient results and identify complications early. Establishing a global collaborative network for data and research sharing will significantly boost collective knowledge and worldwide best practices, ultimately raising the success rate of transplants [49].

AI has the potential to significantly contribute to the progress of regenerative medicine and bioengineering, specifically in areas like organ printing and creating bioartificial kidneys. AI tools can analyze vast amounts of data to pinpoint ideal candidates for regenerative treatments, optimize personalized treatment strategies, and improve the materials and manufacturing techniques used in tissue engineering [50].

**Figure 2:**
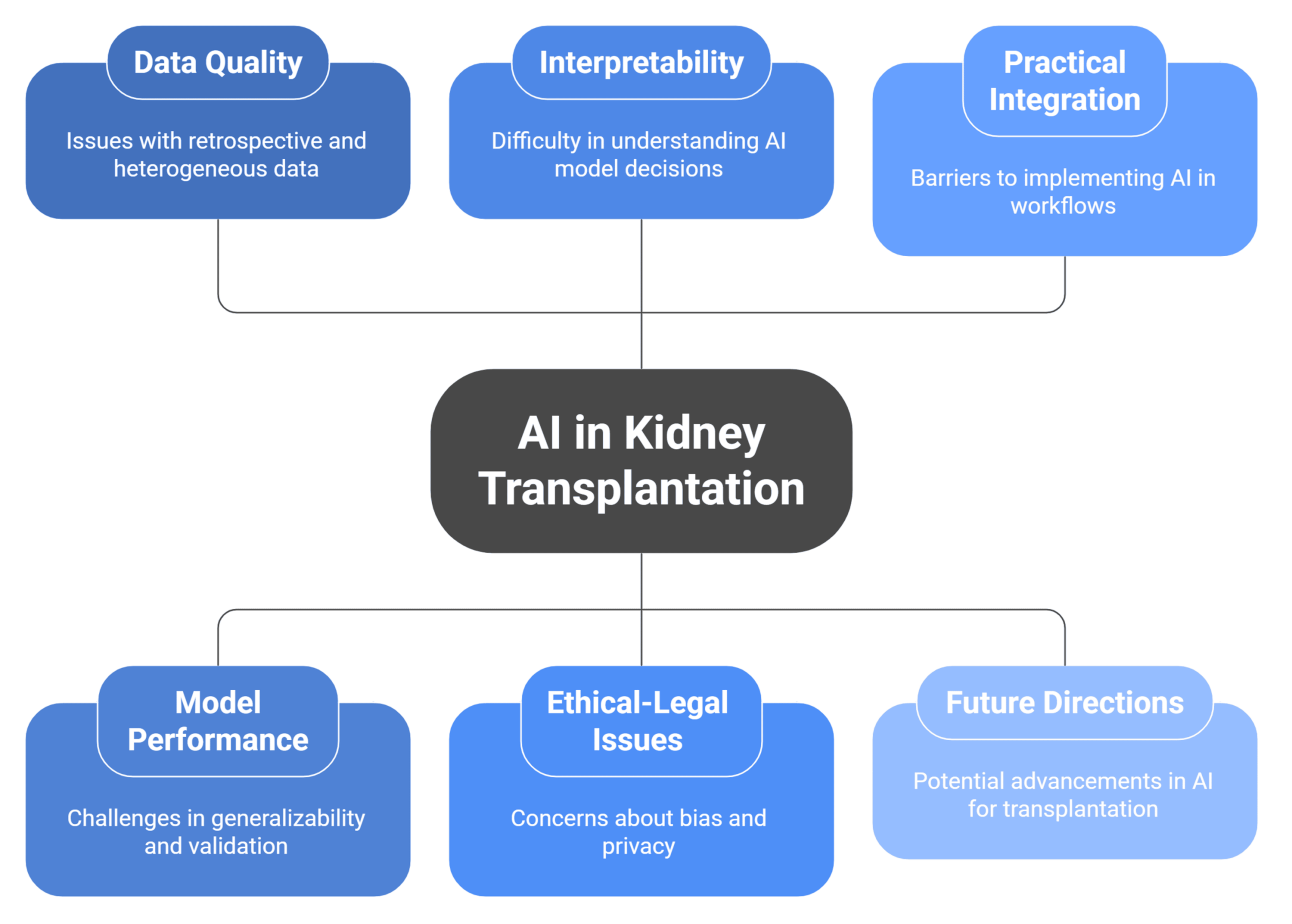
Limitations and future directions A figure created by the article authors based on references [43-51].

AI is poised to transform the machine perfusion process by creating personalized protocols and settings for each kidney, thereby optimizing preservation, organ health, and the likelihood of a successful transplant. A further future possibility involves automated AI-powered monitoring systems that could continuously oversee machine perfusion, identify deviations from ideal conditions, and promptly alert medical staff for intervention. Additionally, AI is expected to soon play a bigger part in the surgical process through new AI robotic surgery platforms (Figure 2) [51].

## Conclusions

The integration of AI and ML into KT over the last decade has significantly enhanced clinical capabilities, ranging from optimized donor allocation to advanced surgical precision. By analyzing complex datasets, AI algorithms have demonstrated superior accuracy in predicting graft survival and rejection compared with traditional statistical methods, while also aiding in the reduction of organ discard rates through better pretransplant matching. Additionally, the emergence of SDS has revolutionized the operating room, utilizing AR and AI-driven robotics to improve anatomical visualization and technical proficiency during procedures.

Despite these promising advancements, the widespread clinical adoption of AI models remains hindered by challenges such as overfitting and selection bias, which stem from a reliance on limited, single-center retrospective data. To overcome these limitations, the future of the field depends on establishing global collaborative networks for data sharing and external validation to ensure that models are robust across diverse populations. Looking ahead, AI is poised to expand its role in regenerative medicine, where it will be instrumental in optimizing bioengineering techniques, such as organ printing and the development of bioartificial kidneys.

## References

[1] Purnell TS, Auguste P, Crews DC (2013). Comparison of life participation activities among adults treated by hemodialysis, peritoneal dialysis, and kidney transplantation: a systematic review. Am J Kidney Dis.

[2] Tonelli M, Wiebe N, Knoll G (2011). Systematic review: kidney transplantation compared with dialysis in clinically relevant outcomes. Am J Transplant.

[3] Siedlecki A, Irish W, Brennan DC (2011). Delayed graft function in the kidney transplant. Am J Transplant.

[4] Nashan B, Abbud-Filho M, Citterio F (2016). Prediction, prevention, and management of delayed graft function: where are we now?. Clin Transplant.

[5] Helfer MS, Pompeo JC, Costa OR, Vicari AR, Ribeiro AR, Manfro RC (2019). Long-term effects of delayed graft function duration on function and survival of deceased donor kidney transplants. J Bras Nefrol.

[6] Jordan MI, Mitchell TM (2015). Machine learning: trends, perspectives, and prospects. Science.

[7] Woodman RJ, Mangoni AA (2023). A comprehensive review of machine learning algorithms and their application in geriatric medicine: present and future. Aging Clin Exp Res.

[8] Sanchez-Martinez S, Camara O, Piella G (2021). Machine learning for clinical decision-making: challenges and opportunities in cardiovascular imaging. Front Cardiovasc Med.

[9] Sarker IH (2021). Machine Learning: algorithms, real-world applications and research directions. SN Comput Sci.

[10] Benjamens S, Dhunnoo P, Meskó B (2020). The state of artificial intelligence-based FDA-approved medical devices and algorithms: an online database. NPJ Digit Med.

[11] Seyahi N, Ozcan SG (2021). Artificial intelligence and kidney transplantation. World J Transplant.

[12] Peng B, Gong H, Tian H, Zhuang Q, Li J, Cheng K, Ming Y (2020). The study of the association between immune monitoring and pneumonia in kidney transplant recipients through machine learning models. J Transl Med.

[13] Santori G, Fontana I, Valente U (2007). Application of an artificial neural network model to predict delayed decrease of serum creatinine in pediatric patients after kidney transplantation. Transplant Proc.

[14] Wittenbrink N, Herrmann S, Blazquez-Navarro A (2019). A novel approach reveals that HLA class 1 single antigen bead-signatures provide a means of high-accuracy pre-transplant risk assessment of acute cellular rejection in renal transplantation. BMC Immunol.

[15] Hummel AD, Maciel RF, Sousa FS (2011). Artificial intelligence techniques: predicting necessity for biopsy in renal transplant recipients suspected of acute cellular rejection or nephrotoxicity. Transplant Proc.

[16] Brier ME, Ray PC, Klein JB (2003). Prediction of delayed renal allograft function using an artificial neural network. Nephrol Dial Transplant.

[17] Hart A, Smith JM, Skeans MA (2018). OPTN/SRTR 2016 annual data report: kidney. Am J Transplant.

[18] Bae S, Massie AB, Luo X, Anjum S, Desai NM, Segev DL (2016). Changes in discard rate after the introduction of the Kidney Donor Profile Index (KDPI). Am J Transplant.

[19] Massie AB, Luo X, Chow EK, Alejo JL, Desai NM, Segev DL (2014). Survival benefit of primary deceased donor transplantation with high-KDPI kidneys. Am J Transplant.

[20] Remuzzi G, Cravedi P, Perna A (2006). Long-term outcome of renal transplantation from older donors. N Engl J Med.

[21] Port FK, Bragg-Gresham JL, Metzger RA (2002). Donor characteristics associated with reduced graft survival: an approach to expanding the pool of kidney donors. Transplantation.

[22] Rao PS, Schaubel DE, Guidinger MK (2009). A comprehensive risk quantification score for deceased donor kidneys: the kidney donor risk index. Transplantation.

[23] Smith JM, Biggins SW, Haselby DG (2012). Kidney, pancreas and liver allocation and distribution in the United States. Am J Transplant.

[24] Schwantes IR, Axelrod DA (2021). Technology-enabled care and artificial intelligence in kidney transplantation. Curr Transplant Rep.

[25] Stegall MD, Stock PG, Andreoni K, Friedewald JJ, Leichtman AB (2017). Why do we have the kidney allocation system we have today? A history of the 2014 kidney allocation system. Hum Immunol.

[26] Bae S, Massie AB, Thomas AG (2019). Who can tolerate a marginal kidney? Predicting survival after deceased donor kidney transplant by donor-recipient combination. Am J Transplant.

[27] Brown TS, Elster EA, Stevens K (2012). Bayesian modeling of pretransplant variables accurately predicts kidney graft survival. Am J Nephrol.

[28] Kilambi V, Bui K, Hazen GB, Friedewald JJ, Ladner DP, Kaplan B, Mehrotra S (2019). Evaluation of accepting kidneys of varying quality for transplantation or expedited placement with decision trees. Transplantation.

[29] Maier-Hein L, Vedula SS, Speidel S (2017). Surgical data science for next-generation interventions. Nat Biomed Eng.

[30] De Backer P, Van Praet C, Simoens J (2023). Improving augmented reality through deep learning: real-time instrument delineation in robotic renal surgery. Eur Urol.

[31] Piana A, Gallioli A, Amparore D (2022). Three-dimensional augmented reality-guided robotic-assisted kidney transplantation: breaking the limit of atheromatic plaques. Eur Urol.

[32] Vedula SS, Ishii M, Hager GD (2017). Objective assessment of surgical technical skill and competency in the operating room. Annu Rev Biomed Eng.

[33] Sriwastwa A, Ravi P, Emmert A (2023). Generative AI for medical 3D printing: a comparison of ChatGPT outputs to reference standard education. 3D Print Med.

[34] Sommer F, Sun B, Fischer J, Goldammer M, Thiele C, Malberg H, Markgraf W (2022). Hyperspectral imaging during normothermic machine perfusion: a functional classification of ex vivo kidneys based on convolutional neural networks. Biomedicines.

[35] Zaza G, Neri F, Bruschi M (2023). Proteomics reveals specific biological changes induced by the normothermic machine perfusion of donor kidneys with a significant up-regulation of latexin. Sci Rep.

[36] Simic-Ogrizovic S, Furuncic D, Lezaic V (1999). Using ANN in selection of the most important variables in prediction of chronic renal allograft rejection progression. Transplant Proc.

[37] Lin RS, Horn SD, Hurdle JF, Goldfarb-Rumyantzev AS (2008). Single and multiple time-point prediction models in kidney transplant outcomes. J Biomed Inform.

[38] Akl A, Ismail AM, Ghoneim M (2008). Prediction of graft survival of living-donor kidney transplantation: nomograms or artificial neural networks?. Transplantation.

[39] Tang H, Poynton MR, Hurdle JF, Baird BC, Koford JK, Goldfarb-Rumyantzev AS (2011). Predicting three-year kidney graft survival in recipients with systemic lupus erythematosus. ASAIO J.

[40] Zhou L, Tang L, Song AT, Cibrik DM, Song PX (2017). A LASSO method to identify protein signature predicting post-transplant renal graft survival. Stat Biosci.

[41] Mark E, Goldsman D, Gurbaxani B, Keskinocak P, Sokol J (2019). Using machine learning and an ensemble of methods to predict kidney transplant survival. PLoS One.

[42] Quinino RM, Agena F, Modelli de Andrade LG, Furtado M, Chiavegatto Filho AD, David-Neto E (2023). A machine learning prediction model for immediate graft function after deceased donor kidney transplantation. Transplantation.

[43] Mizera J, Pondel M, Kepinska M, Jerzak P, Banasik M (2025). Advancements in artificial intelligence for kidney transplantology: a comprehensive review of current applications and predictive models. J Clin Med.

[44] He YJ, Liu PL, Wei T, Liu T, Li YF, Yang J, Fan WX (2025). Artificial intelligence in kidney transplantation: a 30-year bibliometric analysis of research trends, innovations, and future directions. Ren Fail.

[45] Firuzpour F, Pasha AA, Oliaei F, Nasirimehr K, Khosravi M, Rostami G, Saeidnia HR (2025). Artificial intelligence-driven kidney organ allocation: systematic review of clinical outcome prediction, ethical frameworks, and decision-making algorithms. BMC Nephrol.

[46] Knight S (2024). Predicting the future: using AI for clinical support in kidney transplantation. Bull R Coll Surg Engl.

[47] Badrouchi S, Bacha MM, Hedri H, Ben Abdallah T, Abderrahim E (2023). Toward generalizing the use of artificial intelligence in nephrology and kidney transplantation. J Nephrol.

[48] Boenink R, Astley ME, Huijben JA (2022). The ERA Registry Annual Report 2019: summary and age comparisons. Clin Kidney J.

[49] Yang C, Kong G, Wang L, Zhang L, Zhao MH (2019). Big data in nephrology: are we ready for the change?. Nephrology (Carlton).

[50] Nosrati H, Nosrati M (2023). Artificial intelligence in regenerative medicine: applications and implications. Biomimetics (Basel).

[51] Shademan A, Decker RS, Opfermann JD, Leonard S, Krieger A, Kim PC (2016). Supervised autonomous robotic soft tissue surgery. Sci Transl Med.

